# *Prunus persica* Terpene Synthase *PpTPS1* Interacts with PpABI5 to Enhance Salt Resistance in Transgenic Tomatoes

**DOI:** 10.3389/fpls.2022.807342

**Published:** 2022-02-23

**Authors:** Xiangguang Meng, Yuzheng Zhang, Ning Wang, Huajie He, Qiuping Tan, Binbin Wen, Rui Zhang, Mingyue Sun, Xuehui Zhao, Xiling Fu, Dongmei Li, Wenli Lu, Xiude Chen, Ling Li

**Affiliations:** ^1^College of Horticulture Science and Engineering, Shandong Agricultural University, Tai’an, China; ^2^State Key Laboratory of Crop Biology, Shandong Agricultural University, Tai’an, China; ^3^Shandong Collaborative Innovation Center for Fruit & Vegetable Production With High Quality and Efficiency, Shandong Agricultural University, Tai’an, China

**Keywords:** peach, *PpTPS1*, ABA, salt stress, *PpABI5*

## Abstract

Terpene synthase (*TPS*) is related to the production of aromatic substances, but there are few studies on the impact of abiotic stress on *TPS* and its molecular mechanism, especially in peaches. This study found that salt resistance and abscisic acid (ABA) sensitivity of transgenic tomatoes were enhanced by overexpression of *PpTPS1*. Moreover, it was found that *PpTPS1* interacted with and antagonized the expression of the *bZIP* transcription factor *ABA INSENSITIVE 5* (*PpABI5*), which is thought to play an important role in salt suitability. In addition, *PpTCP1*, *PpTCP13*, and *PpTCP15* were found to activate the expression of *PpTPS1* by yeast one-hybrid (Y1H) and dual-luciferase assays, and they could also be induced by ABA. In summary, *PpTPS1* may be involved in the ABA signaling regulatory pathway and play an important role in salt acclimation, providing a new reference gene for the improvement of salt resistance in peaches.

## Introduction

China is a prominent country in the global peach industry, and peach fruit tree cultivation is an important industry for improving the disposable income of the farmers (Deng et al., 2018). Salt damage is an important issue affecting the development of global agriculture in the 21st century, and the saline land area in China is as high as 99 million hm^2^. As a salt-sensitive, non-saline plant, peach trees can be seriously affected by salt stress in terms of growth, development, fruit yield, and quality of fruit trees (Munns, 2011; Zhu et al., 2018). With bioengineering and gene assembly technology techniques, genetic transformation of salt tolerance genes and molecular biomarker breeding have become important means of improving salt tolerance in plants (Sun et al., 2018). With this process, it has become particularly important to screen excellent, salt-tolerance candidate genes and study their molecular regulatory networks (Nayyeripasand et al., 2021).

Terpene synthases (*TPSs*) are key enzymes for terpene formation, and use isopentenyl diphosphates of different lengths as substrates. Most plant *TPSs* have multiple substrates and exhibit catalytic activity, which is an important reason for the rich variety of terpenoids (Chen et al., 2011). *TPSs* generally have 550–850 amino acid residues, including a C-terminal active structural domain and an N-terminal structural domain (Bohlmann et al., 1998; Chen et al., 2003; Dudareva et al., 2003). Leesburg found that almost all *TPSs* have a conserved aspartic acid-rich structural domain DDXXD, which plays an important role in the binding of divalent metal ions to substrates (Lesburg et al., 1997). Current studies on *TPSs* have focused on aroma quality and bio-resistance, and they showed transfer of linalool synthase from fanwort in tomatoes to increase the linalool content in ripe fruits (Lewinsohn et al., 2001); overexpression of geraniol synthase from lemon in tomato was shown to increase the geraniol content (Davidovich-Rikanati et al., 2007); and limonene synthase inhibition in citrus was shown to enhance the resistance of navel orange fruit to Penicillium infestation (Rodriguez et al., 2011, 2014). However, there are few studies on the impact of abiotic stress on *TPSs*. Mafu et al. (2018) performed oxidative stimulation experiments on maize roots with 1 mM CuSO_4_ artificially simulating abiotic stress factors and found that dolabralexin-like diterpene accumulated continuously in the roots for 48 h. *ZmTPS6* could respond to abscisic acid (ABA) signaling, and overexpression of *ZmTPS6* increased drought resistance in maize (Hou et al., 2017; Lee, 2018). The transcription factors *FhMYB21* and *FhMYC2* of the *MYB* and *MYC* families, which are thought to be associated with biotic stress, regulate the expression of *FhTPS1* in *A. fragrans* (Yang et al., 2020). Peach *TPS1* was associated with the synthesis of linalool (Liu et al., 2017), so determining whether it is involved in abiotic stresses was of interest.

Abscisic acid can act as a stress hormone (Larosa et al., 1985; Leung and Giraudat, 1998), and under salt stress, ABA can alleviate and mitigate high salt damage to the plant body by inducing a large accumulation of osmolytes in the plant (Shahid et al., 2015; Sharma et al., 2019). It has been demonstrated that ABA alleviates salt stress by regulating target proteins through a series of signal transduction pathways, including ABA receptor recognition, intracellular signaling, and phosphorylation of corresponding transcription factors, to regulate the expression of related genes (Sreenivasulu et al., 2012; Fujii, 2014). *ABI5* is a class of *bZIP* transcription factors downstream of ABA, which can be activated by phosphorylation and involved in stress regulation (Dai et al., 2013). Exogenous application of ABA or stress conditions such as high salt and drought can lead to the induction of *ABI5* expression (Lopez-Molina et al., 2001, 2010). Coexpression of *Arabidopsis ABI3* and *ABI5* in cotton was able to improve cotton resistance to drought stress by regulating reactive oxygen species cluster scavenging and the expression of osmoregulatory marker genes (Mittal et al., 2014). In addition, studies of rice and wheat have also revealed that *ABI5* is associated with the plant response to adversity stress (Yang et al., 2010, 2011). *ABI5* is involved in the biological process of the plant response to adverse stress and plays an important role in the process of plant adaptation to salt.

In the present study, we overexpressed *PpTPS1* in tomatoes and found that ABA sensitivity and resistance were enhanced in transgenic tomatoes. We verified that *PpTPS1* interacts with *PpABI5* and that *PpTCP1*, *PpTCP13*, and *PpTCP15* can activate the expression of *PpTPS1*, and hence we hypothesized that *PpTCP1*, *PpTCP13*, *PpTCP15*, and *PpTPS1* play important roles in salt adaptation and their functions are closely related to the ABA signaling pathway.

## Materials and Methods

### Plant Materials

Beginning in September 2019, samples of the fruit, roots, leaves, flowers, and stems of a 6-year-old “Chun Xue” peach tree were obtained from the Horticulture Experimental Station of Shandong Agricultural University. Tissue samples were stored at −80°C before RNA extraction.

The transgenic tomato variety was “Micro Tom” purchased from Nanjing Fengtai Horticultural Seed (Nanjing, China) Co. Tomato seeds were sterilized, soaked in warm water, germinated on Murashige and Skoog (MS) medium for 3 days, and then used for Agrobacterium infection. The seeds used in the germination rate experiment were treated in the same way. Annual hickory peach seedlings were provided by the National Apple Engineering Center Laboratory. A 3:1:1 mixture of grass charcoal, vermiculite, and perlite was used as a substrate for each tomato material and annual peach seedling planted in a 12-cm-wide square plastic culture bowl. Tomato and annual peach seedlings were cultivated in a growth chamber in which the temperature, relative humidity, photoperiod, and photosynthetic photon flux density were 26°C/20°C, 65%, 14 h/10 h (day/night), 300 μmol m^–2^ s^–1^, respectively. The leaves of the annual peach seedlings were sampled in separate periods after spraying with 100 μM ABA, at 4°C, root soaking with 10% PEG, and watering with 150 mM NaCl. Thirty milliliters of 150 mM NaCl solution was applied to the transgenic tomato substrate every afternoon and the wild type (WT) was treated with water for 12 days. The leaves of transgenic tomatoes and the WT were then taken for physiological data analysis.

### RNA Extraction and qRT-PCR Analysis

RNA from the samples was extracted using the RNA Pure Plant Kit (DNase I) (CW0559) and first strand cDNA was obtained using the SuperRT cDNA Synthesis Kit (CW0741) for qRT-PCR. An Ultra SYBR Mixture Kit (CW0957) was used for qRT-PCR analysis, and the primers are listed in Supplementary Table 1. *PpActin* (*Prupe.6G163400*) is an internal reference gene of peach (Zhao et al., 2020), and *SlAction* (*Solyc11g005330*) is the internal reference gene of tomato (Hu et al., 2021). These kits were purchased from Kangwei Century Biotechnology (Jiangsu) Co., and primer synthesis was performed by Biotech Bioengineering (Shanghai) Co.

### Cloning and Overexpression of *PpTPS1*

Primers (5′- ATGGCATTGTTTTCTATGGCCA-3′; 5′- CACAGATTCAGTTTCATACAGCATCG-3′) were designed by searching the NCBI database using the cDNA sequence *Prupe.4G030400* (*PpTPS1*). The amplification reaction was performed using the cDNA of “Chun Xue” peach as the template, and the amplified products were sequenced and compared with the NCBI database. *PpTPS1* was cloned into the plant expression vector PBI121-GFP using *Sma*I single enzyme digestion (Supplementary Figure 1). The recombinant plasmid was transformed into GV3101 *Agrobacterium* using the freeze-thaw method, and the transformed *Agrobacterium* was then used to infect the “Micro Tom” tomato explants (Jian et al., 2019). After induction of germination, rooting, and identification, overexpressed transgenic tomatoes were obtained.

### Determination of Relative Water Content, and Relative Electrical, Malondialdehyde, Proline, Soluble Protein, and Soluble Sugar Contents

Tomato leaves were taken after treatment to measure the relative water content, relative electrical measurements, and MDA contents, and the analysis was performed as described previously by Roger (2001). The proline, soluble protein, and soluble sugar contents were measured in the treated tomato leaves, according to Zhuang et al. (2019).

### Virus-Induced Gene Silencing Transiently Infests Peach Fruit

NCBI was used to design specific primers for *PpABI5* (5′-GAGAGTGGTGAATGGGGGTG-3′; 5′-ACCTTTTCCACCGGTCCATC-3′), ligate the amplified *PpABI5* fragment with the TRV2 plasmid using restriction enzymes *Eco*RI and *Bam*HI, and then transfer it into the *Agrobacterium* GV3101. The PpABI5-TRV2 and TRV2 *Agrobacterium* liquid were mixed with the *Agrobacterium* liquid of TRV1 in a 1:1 ratio, and they were injected into the green ripe peach fruit, followed by vacuum treatment for 10 min and dark treatment for 1 day. The sample was then left under normal lighting condition for 3 days. The pulp was used as a sample for qRT-PCR analysis (Zhang et al., 2015).

### Yeast Two-Hybrid Assays

The peach yeast two-hybrid library was established. Then PpTPS1-pGBKT7 vector was constructed and transferred it into Y2H and cultured on -Trp, -Trp/X-α-gal medium at 30°C to verify the autonomous activation activity. Afterward, the library was initially screened for interacting genes, and the screened *PpABI5* (*Prupe.7G112200.1*) was inserted into the pGADT7 vector (Zhang et al., 2021). The Matchmaker Yeast Two-Hybrid System User Manual was followed to perform mutual verification again.

### Pull-Down Assays

*PpABI5* was cloned into a pGEX-6P-1 vector containing a GST tag, and *PpTPS1* was cloned into a pET32a vector containing a His-tag. The vectors were transformed into BL21 DE3 Escherichia coli, and 1 mM isopropyl β-D-thiogalactoside (IPTG) was used to induce the expression of the fusion protein in the bacteria on a shaker at 37°C (Zhao et al., 2020). After the induced PpTPS1-His fusion protein and PpABI5-GST protein were purified by dialysis, the protein was mixed according to the combination of PpTPS-His/GST and PpTPS-His/PpABI5-GST, purified by a GST purification column, and then subjected to Western blot analysis. The GST/His-tagged Protein Purification Kit (P2262/P2229), His/GST Tag Mouse Monoclonal Antibody (AF5060/AF5063), and goat anti-mouse IgG (A0216) used in the experiment were purchased from Shanghai Biyuntian Biotechnology Co.

### Bimolecular Fluorescence Complementation Assays

The PpABI5-pSPYNE and PpTPS1-pSPYCE recombinant plasmids were constructed and transformed into *Agrobacterium tumefaciens* GV3101. The two bacterial solutions were mixed at a 1:1 ratio to transfect onion epidermal cells for 30 min, and PpABI5-pSPYNE + pSPYCE and PpTPS1-pSPYCE + pSPYNE, 1:1 mixed bacterial liquid, were used as the two groups of controls (Chen et al., 2018). After 48 h of culture in MS medium, the cells were observed with a laser-scanning confocal microscope (LSM880).

### Yeast One-Hybrid Assays

*PpTPS1^100–400^* (primers: 5′-AAAGGCAGAGCCATGCTTAG-3′; 5′-TAAAGAGACCCTCGCGGGCT-3′) was amplified using 5′−100 to −400 bp genomic DNA of the 2-kb upstream promoter of *PpTPS1* as a template. FastDigest BstBI was used to linearize the plasmid, transform it into yeast one-hybrid (Y1H) Gold, and detect the lowest concentration of AbA that inhibits the growth of bait yeast strains on Ura medium at 30°C. Next, the lowest concentration of AbA was used for the Y1H screening library (Wang et al., 2020). The screening results were sequenced and compared with the sequences in NCBI.

### Dual-Luciferase Assays

*PpTCP1*, *PpTCP13*, and *PpTCP15* were inserted into the pGreenII0029 62-SK vector, and the promoter fragment PpTPS1^100–400^ was inserted into the pGreenII0800-Luc vector (Walter et al., 2004). The vectors were then transformed into *A. tumefaciens* strain GV3101 pSoup. A mixture of four Agrobacterium strains was injected on the back of tobacco leaves: PpTCP1/PpTCP13/PpTCP15-62SK + PpTPS1-Luc, PpTCP1/PpTCP13/PpTCP15-62SK + Luc, and 62SK + PpTPS1-Luc. After 2–3 days, 1 mM D-luciferin and sodium salt were applied to the backs of the tobacco leaves, and an *in vivo* imaging system was used to detect fluorescence.

### Statistical Analyses

Biological triplicates were performed for each sample, and the data are expressed as the mean ± standard error (SE). Two-way ANOVA was performed using SPSS for Windows 24 when applicable. The qRT-PCR data were analyzed by Duncan’s test. A significance level of *P* < 0.05 was used.

## Results

### *PpTPS1* Response to Abiotic Stress and Expression Analysis in Different Tissues

**FIGURE 1:**
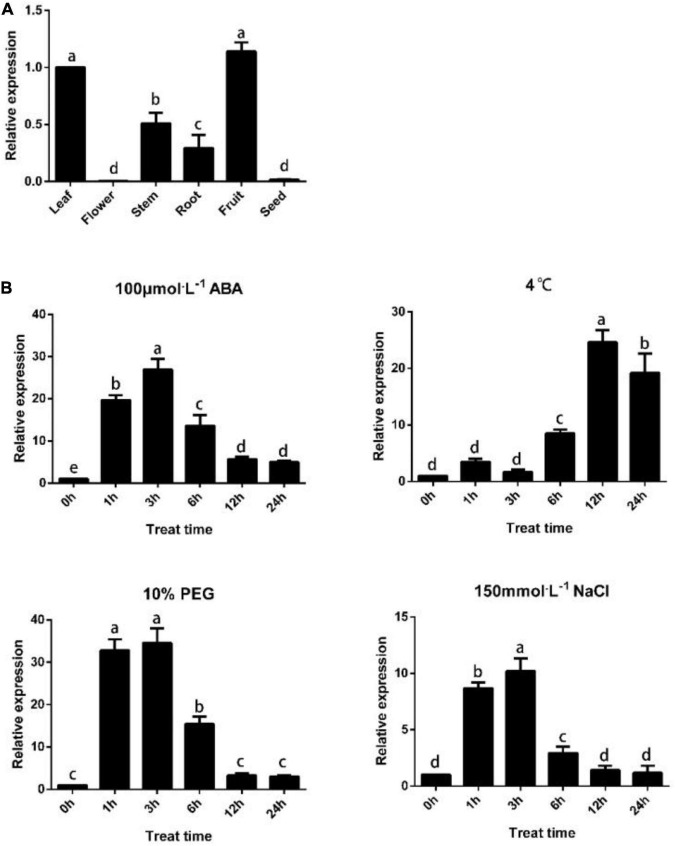
*PpTPS1* tissue specificity analysis and expression pattern analysis under abiotic stress. **(A)** PpTPS1 tissue specificity analysis. **(B)** Annual peach leaves treated with 100 μM ABA, 4°C, 10% PEG and 150 mM NaCl for 0, 1, 3, 6, 12, and 24 h were used as samples to analyze the expression of the *PpTPS1* model. Biological triplicates were performed for each sample. Different letters represent significant differences according to ANOVA and Duncan’s test (*P* < 0.05).

The leaves, flowers, stems, roots, fruit, and seeds of 6-year-old “Chun Xue” peach trees were used as samples to perform qRT-PCR analysis to study the expression patterns of *PpTPS1* in different tissues of peaches. The results showed *PpTPS1* had the highest expression in fruit, followed by leaves, and the lowest expression was observed in flowers, indicating that *PpTPS1* may be widely involved in a variety of physiological activities in plants (Figure 1A).

Most *TPSs* genes contain abundant promoter *cis-*elements related to abiotic stress, but no further research has been conducted. To initially explore whether *PpTPS1* is induced by abiotic stress, we analyzed its expression pattern under exogenous ABA, low-temperature, salt, and drought treatments. The results showed that the target gene was significantly induced under the treatment of 100 μM ABA, 4°C, 10% PEG, and 150 mM NaCl. Interestingly, the expression pattern of *PpTPS1* under NaCl and PEG treatment was similar to that under ABA treatment, and its expression level increased rapidly at first, reached a maximum at 3 h and then decreased (Figure 1B). This result indicated that *PpTPS1* could play a role in most abiotic stresses.

### Functional Identification of *PpTPS1*

**FIGURE 2:**
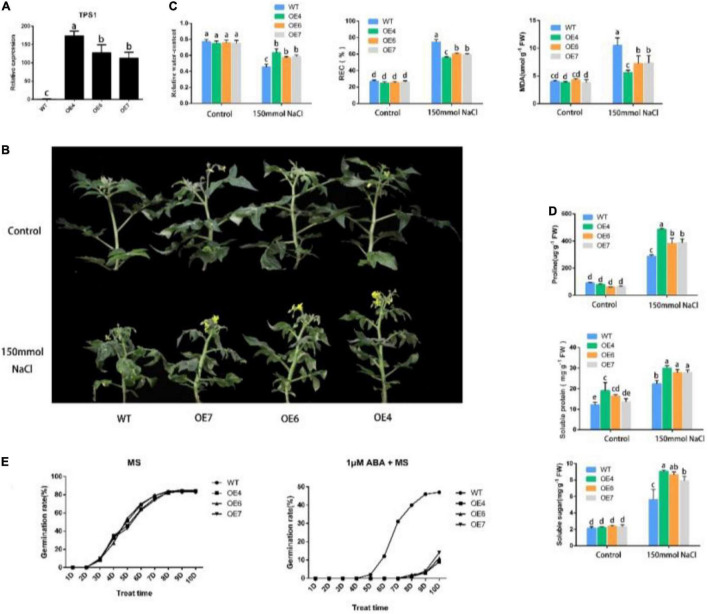
Functional identification of *PpTPS1*. **(A)** qRT-PCR analysis of *PpTPS1* transgenic tomato lines under normal condition. Transgenic tomato lines under normal condition. **(B)** Growth status of WT and transgenic tomatoes treated with NaCl for 12 days. **(B,C)** The leaves of WT and transgenic tomatoes treated with NaCl for 12 days were taken to measure the relative electrical, and MDA contents Growth status of WT and transgenic tomatoes treated with NaCl for 12 days. **(C)** Analysis of the relative electrical measurement and MDA content and relative water content of WT and transgenic tomatoes treated with NaCl for 12 days. **(E)** Analysis of the seed germination rate of WT, OE4, OE6, OE7 on MS and MS + 1 μM ABA media for 10 days. Each line contained 100 seeds. Biological triplicates were performed for each sample. Different letters represent significant differences according to ANOVA and Duncan’s test (*P* < 0.05).

The amplification products of “Chun Xue” were sequenced and compared with the NCBI, and the sequences were consistent with *PpTPS1* (Genbank ID: OL855977). The results of RT-PCR (Supplementary Figure 2) and qRT-PCR analyses, showed that *PpTPS1* was successfully overexpressed in tomatoes, and three overexpressing tomato lines were obtained (Figure 2A). We treated transgenic and WT “Micro TOM” tomatoes that were grown for 5 weeks and grew consistently with 150 mmol⋅L^–1^ NaCl solution for 12 days, and the control group was treated with water. As shown in Figure 2B, the leaves of WT tomatoes treated with NaCl showed curling, yellowing, and necrosis, while the transgenic tomatoes grew better, and the higher the expression of *PpTPS1* was, the better the plants grew; the control group exhibited no significant difference. We measured the relative water content of transgenic and WT tomatoes after salt treatment, and the results demonstrated that the relative water content of transgenic tomatoes was significantly higher than that of WT tomatoes. The relative electrical and malondialdehyde (MDA) content measurements showed that WT tomatoes suffered significantly more damage than transgenic tomatoes, and the trend was consistent with the expression level (Figure 2C). This result suggests that salt treatment may cause a relatively large disruption of the cellular structure of WT tomatoes compared with transgenic tomatoes, disrupting the ionic balance, but the exact mechanism needs further experimental verification.

Content of osmotic adjustment substances can be used as an evaluation index to measure salt tolerance. Numerous studies have shown that plants can increase their resistance to abiotic stress by increasing the content of osmotic adjustment substances. To preliminarily explore the mechanism of *PpTPS1* transgenic tomatoes in response to salt stress, we measured the content of its osmotic adjustment substances. The results showed that the proline, soluble protein, and soluble sugar contents of transgenic tomatoes after NaCl treatment were significantly higher than those of the WT tomatoes, especially OE4 (Figure 2D). Therefore, we speculate that the increased content of osmotic regulators may be one reason transgenic tomatoes have better salt resistance.

To explore whether *PpTPS1* responds to ABA, we analyzed the seed germination rate of PpTPS1-overexpressing tomatoes under ABA treatment. The results showed that there was no significant difference between the seed germination rate of WT and transgenic tomatoes on a normal MS medium. On 1 μM ABA + MS medium, the seed germination rate of transgenic tomatoes was significantly lower than that of the WT (Figure 2E), and it was opposite to the expression trend. This result shows that overexpression of *PpTPS1* can increase the sensitivity of plants to ABA.

### *PpTPS1* Interacts With *PpABI5*

**FIGURE 3:**
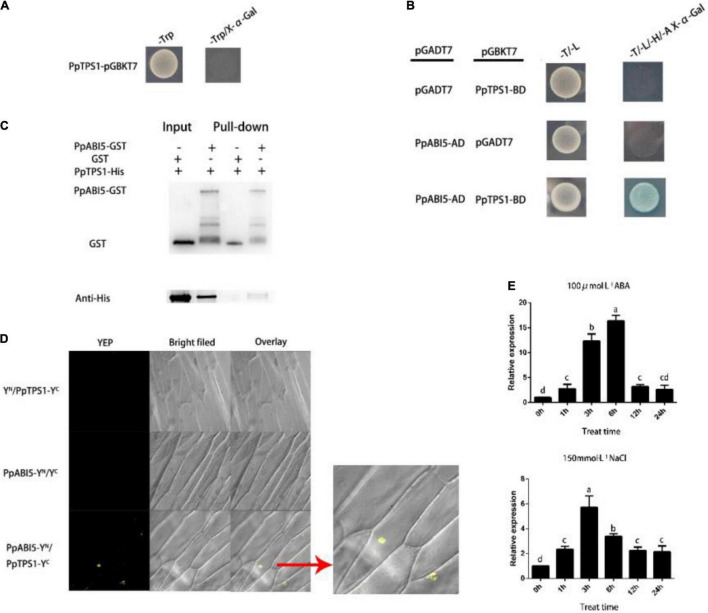
*PpABI5* interacts with *PpTPS1* and responds to salt stress. **(A)** *PpTPS1* has no autonomous activation activity. **(B)** Verification of the interaction between *PpTPS1* and *PpABI5* by a yeast two-hybrid (Y2H) experiment. Yeast containing PpTPS1-BD and PpABI5-AD can grow on DDO (SD/-Leu/-Trp), and they can also grow and turn blue on QDO (SD-Leu/-Trp/-His/-Ade/X-α-Gal). **(C)** GST pull-down analysis verified the interaction of *PpTPS1* and *PpABI5*. GST, PpTPS1-His, and PpABI5-GST proteins were all purified once before the experiment. **(D)** Bimolecular fluorescence complementation (BiFC) verification of the interaction between *PpTPS1* and *PpABI5*. **(E)** Annual peach leaves treated with 100 μM ABA and 150 mM NaCl for 0, 1, 3, 6, 12, and 24 h were used as samples to analyze the expression of the *PpABI5* model. Biological triplicates were performed for each sample. Different letters represent significant differences according to ANOVA and Duncan’s test (*P* < 0.05).

After testing for autonomous activation activity, we found PpTPS1 had no autonomous activation activity (Figure 3A). We used PpTPS1-BD as the “bait” to screen its interacting proteins in the library. From the preliminary screening results (Supplementary Table 2), we found that *PpABI5* may interact with *PpTPS1*, and previous studies of *ABI5* have shown that it is involved in salt stress. We cloned the full-length *PpABI5*, constructed the PpABI5-AD vector, and cotransformed Y2H competent yeast with PpTPS1-BD. The results showed that *PpTPS1* can interact with *PpABI5* (Figure 3B). To further verify the interaction between *PpTPS1* and *PpABI5*, we conducted *in vitro* protein interaction verification (pull-down) and bimolecular fluorescence complementation (BiFC) analysis (Figures 3C,D), and the results supported the above conclusions.

To verify whether *PpABI5* responded to salt stress, we analyzed the expression pattern of *PpABI5* after exogenous ABA and NaCl treatment. The results showed that *PpABI5* can be induced by salt stress, which is consistent with our hypothesis and the results of previous studies (Figure 3E).

### There Is an Antagonistic Relationship Between *PpTPS1* and *PpABI5*

**FIGURE 4:**
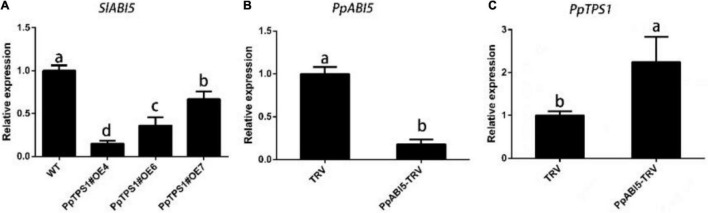
Analysis of the antagonistic relationship between *PpTPS1* and *PpABI5*. **(A)** Analysis of the expression of the *PpABI5* homologous gene *SlABI5* in three transgenic tomato lines, OE4, OE6, and OE7. **(B)** The expression level of *PpABI5* after 4 days of transient silencing of *PpABI5* in peach fruit by the VIGS system using TRV as a vector. **(C)** Analysis of *PpTPS1* expression after transient silencing of *PpABI5*. Biological triplicates were performed for each sample. Different letters represent significant differences according to ANOVA and Duncan’s test (*P* < 0.05).

Because of the interaction between *PpTPS1* and *PpABI5*, we were curious about the expression of *SlABI5* in *PpTPS1*-overexpressing tomatoes. The qRT-PCR results showed that the expression of *SlABI5* in transgenic tomatoes was significantly lower than that in WT tomatoes (Figure 4A). Then we constructed the PpABI5-TRV2 vector and successfully silenced *PpABI5* in peach fruit (Figure 4B). The qRT-PCR results showed that the expression of *PpTPS1* in peach fruit was significantly increased after *PpABI5* was silenced compared with the control (Figure 4C). This result indicated that there is an antagonistic relationship between *PpTPS1* and *PpABI5.*

### *PpTCP1*, *PpTCP13*, and *PpTCP15* Regulate the Expression of *PpTPS1*

**FIGURE 5:**
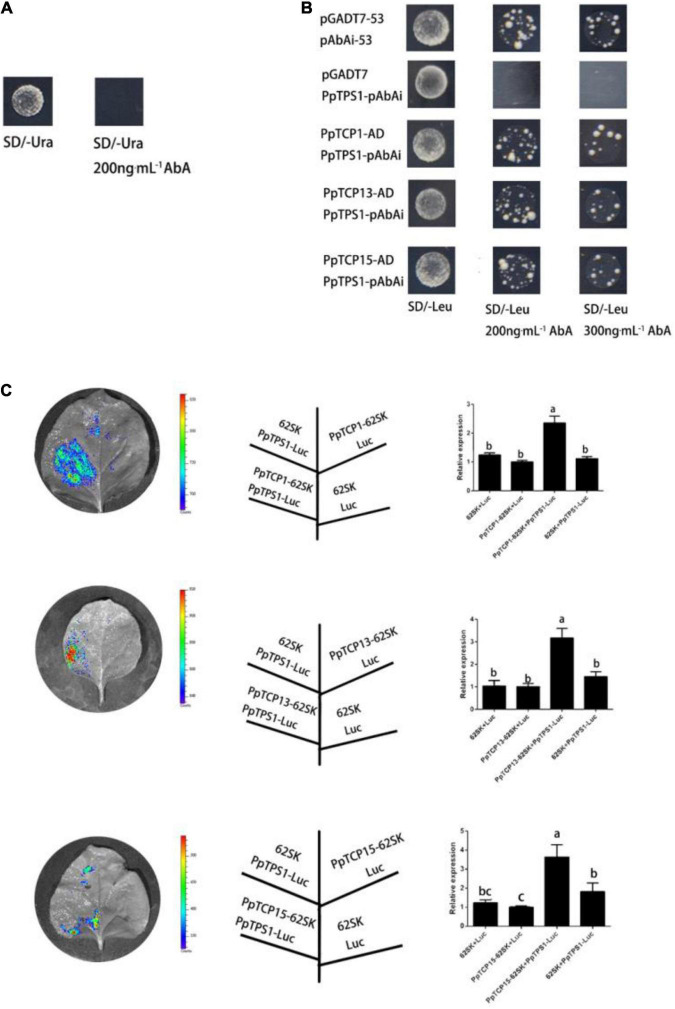
*PpTCP1*, *PpTCP13*, and *PpTCP15* can activate the expression of *PpTPS1*. **(A)** The PpTPS1-pAbAi “bait” strain was inhibited by 200 ng⋅mL^–1^ AbA. **(B)** The PpTCP1-pGADT7, PpTCP13-pGADT7, and PpTCP15-pGADT7 plasmids were constructed, transformed into Y1H medium containing PpTPS1^100–400^-pAbAi, and cultured in Leu medium at 30°C in the dark for 3 days. The grown yeast was then diluted with 0.9% NaCl solution (OD600 = 0.002), spotted on SD/-Leu at concentrations of 200 and 300 ng⋅mL^–1^ AbA, and cultured in the dark at 30°C for 3–5 days. **(C)** A tobacco dual-luciferase experiment verified that *PpTCP1*, *PpTCP13*, and *PpTCP15* could activate the expression of *PpTPS1*. Biological triplicates were performed for each sample. Different letters represent significant differences according to ANOVA and Duncan’s test (*P* < 0.05).

To explore the regulatory relationship of *PpTPS1* upstream genes, we analyzed the 2-kb promoter sequence upstream of ATG, the translation start site of the *PpTPS1* gene, and found that there were multiple TCP-binding motifs, G (T/C) GGNCCCAC motifs, in the 118–378 bp fragment of the promoter (Cubas, 2002; Kosugi and Ohashi, 2002; Li et al., 2005). Previous studies revealed that signal transmission between *TCP* transcription factors and ABA played an important role in salt stress (Tatematsu et al., 2008). The experiments in the present study showed that the use of 200 ng mL^–1^ AbA can inhibit the growth of the PpTPS1^100–400^-pAbAi “bait” yeast strain (Figure 5A). A Y1H screen library was performed using PpTPS1^100–400^-pAbAi as bait, and the initial screening results contained three *TCP* family genes: *PpTCP1* (*Prupe.3G240200.1*), *PpTCP13* (*Prupe.3G252600*.1), and *PpTCP15* (*Prupe.5G191200*.1) (Supplementary Table 3). Further verification revealed that the PpTPS1^100–400^-pAbAi “bait” yeast transferred into PpTCP1-AD, PpTCP13-AD, and PpTCP15-AD plasmids grew normally on SD/-Ura-Leu/300 ng⋅mL^–1^ AbA, indicating that *PpTCP1*, *PpTCP13*, and *PpTCP15* could bind to the *PpTPS1* promoter (Figure 5B).

To explore how *PpTCP1*, *PpTCP13*, and *PpTC15* regulate the expression of *PpTPS1*, we performed a dual-luciferase assay. The results showed that the fluorescence intensity of the regions where PpTPS1^100–400^-Luc coexpressed with PpTCP1-62SK, PpTCP13-62SK, and PpTCP15-62SK increased significantly (Figure 5C), indicating that *PpTCP1*, *PpTCP13*, and *PpTCP15* could promote the expression of *PpTPS1*.

### *PpTCP1*, *PpTCP13*, and *PpTCP15* Can Be Induced by Abscisic Acid

**FIGURE 6:**
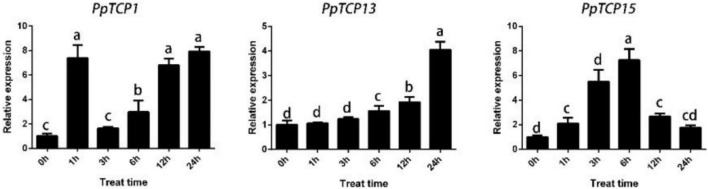
Expression patterns of *PpTCP1*, *PpTCP13*, and *PpTCP15* after ABA treatment. One-year-old peaches were sprayed with 100 μM ABA, and samples were taken at 0, 1, 3, 6, 12, and 24 h for qRT-PCR analysis. Biological triplicates were performed for each sample. Different letters represent significant differences according to ANOVA and Duncan’s test (*P* < 0.05).

We analyzed the 1.5 kb promoter region upstream of *PpTCP1*, *PpTCP13*, and *PpTCP15*, and the results showed that these three genes all contained the *cis-*acting element ABRE, which is involved in the ABA response (Table 1). We then performed qRT-PCR analysis on peach seedlings treated with ABA. As shown in Figure 6, ABA significantly induced the expression of *PpTCP1*, *PpTCP13*, and *PpTCP15*, but the expression patterns of the three were different. We speculated that these genes could play different roles in the process of ABA signaling.

**TABLE 1:** Analysis of *cis-*acting elements of *PpTCP1, PpTCP13*, and *PpTCP15.*

Genes	*Cis-*acting element	Numbers	Sequence	Annotation
*PpTCP15*	ARE	6	AAACCA	anaerobic induction
	ABRE	2	CACGTG	abscisic acid-responsive
	AuxRR-core	1	GGTCCAT	auxin-responsive
*PpTCP13*	TCA-element	1	TCAGAAGAGG	salicylic acid responsive
	GARE-motif	1	TCTGTTG	gibberellin responsive
	ABRE	2	ACGTG	abscisic acid-responsive
	ARE	1	AAACCA	anaerobic induction
	TC-rich repeats	1	ATTCTCTAAC	defense and stress-responsive
	LTR	1	CCGAAA	low-temperature responsive
*PpTCP1*	ABRE	1	ACGTG	abscisic acid-responsive
	CGTCA-motif	1	CGTCA	MeJA-responsiveness
	TGACG-motif	1	TGACG	MeJA-responsiveness
	ARE	2	AAACCA	anaerobic induction

## Discussion

Plants perceive biotic and abiotic stress signals and initiate a series of hormonal signaling pathways that regulate the expression of downstream defense genes to generate relevant defense responses. Among these hormones, ABA plays an important role in the defense against abiotic stresses such as drought, high salt, cold, high temperature, and mechanical damage (Zhang et al., 2006; Lata and Prasad, 2011). Many studies have shown that an increase in ABA content plays an indispensable role in improving the salt tolerance of plants. The specific role of ABA is to relieve osmotic stress and ion stress caused by excessive salt, maintain water balance, and protect the integrity of the cell membrane structure. The accumulation of osmotic regulators, such as proline, soluble protein, and soluble sugar, can alleviate the damage caused by abiotic stress and improve plant tolerance. Under salt stress, ABA can induce a large accumulation of proline, an osmotic regulator in plants, and increase the activity of related protective enzymes (Espasandin et al., 2014). Salt stress-related genes can regulate osmotic substances by participating in ABA signal transduction to achieve their related functions. *GhMYB73* can positively regulate ABA signaling, increase the proline content, and enhance salt tolerance in transgenic cotton (Zhao et al., 2019). *WRKY* family transcription factors such as *GhWEKY17*, *GhWRKY41*, *ZmWRKY33*, and *DoWRY5* can regulate the ABA metabolic pathway, participate in ABA signal transmission, and, thus, participate in the regulation of osmotic stress (Li et al., 2013; Yan et al., 2014; Chu et al., 2016). In this study, we found that *PpTPS1* can be induced by exogenous ABA, and the seed germination rate experiment of transgenic tomato showed that *PpTPS1* enhanced the sensitivity of plants to ABA (Figure 2E). The relative water content, relative electrical measurement, and MDA content of transgenic plants after salt stress treatment were lower than those of WT plants, while the content of osmoregulatory substances was significantly higher than that of WT plants. Therefore, we speculate that *PpTPS1* increases plant salt resistance by participating in the ABA signaling pathway and, thus, increasing the accumulation of osmotic substances. *ABI5* is involved in the biological process of the plant’s response to adverse stress. Plants with *OsABI5* overexpression exhibited faster expansion loss, yellowing, and growth inhibition under salt stress, while *OsABI5*-antisense plants were salt tolerant (Zou et al., 2007). Transgenic tobacco *ZmABI5* showed reduced chlorophyll content, proline accumulation, and antioxidant enzyme activity under drought, salt, heat, and cold stresses (Yan et al., 2012). *ABI5* also plays an important role in the feedback regulation of ABA core signaling and ABA biosynthesis. In Arabidopsis, Wang found that *ABI1 ABI2*, encoding a PP2C phosphatase that negatively regulates ABA signaling, is directly activated by *ABI5* (Wang et al., 2019). In barley *HvSnRK2.1*, *HvPP2C4* and the key ABA biosynthetic enzyme *HvNCED1* are activated in the *hvabi5* synapse (Zhu et al., 2020). We verified that *PpTPS1* interacted with *PpABI5* by Y2H, GST pull-down, and BiFC assays, and consistent with expectations, *PpABI5* expression was also induced by ABA and salt stress. A qRT-PCR analysis of transgenic tomato and peach TRV samples revealed that *PpTPS1* and *PpABI5* antagonized each other (Figures 3, 4). Therefore, we speculated that *PpABI5* may play a negative regulatory role during salt stress in peach, and *PpTPS1* can be positively involved in ABA signaling by ABA activation, while *PpTPS1* can enhance ABA biosynthesis and signaling to enhance salt resistance of plants by antagonizing *PpABI5* expression. However, further experimental evidence is still required.

*T* encode plant-specific transcription factors containing a bHLH motif that can bind to DNA or produce protein–protein interactions. Previous studies have revealed that *TCPs* can participate in ABA regulatory pathways and play an important role in abiotic stresses. In Arabidopsis, *DOF6* is an ABA biosynthesis gene, and *AtTCP14* can interact with its target *DOF6* to inhibit ABA biosynthesis (Tatematsu et al., 2008). Under stress, rice *OsTCP19* causes significant upregulation of the ABA biosynthesis gene *ABI4*; overexpression of *OsTCP19* decreases water loss and reactive oxygen species (ROS) in the plant, thereby improving plant salinity tolerance (Mukhopadhyay and Tyagi, 2015); *OsPCF6* and *OsTCP21* improve plant tolerance to cold stress by altering ROS scavenging capacity Zhou et al. (2013); and (Wang et al., 2014) found that miR319-targeted *AtTCP14* improves plant tolerance during drought and salt stress. The upstream region of the *PpTPS1* promoter contains multiple TCP binding sites G (T/C) GGNCCCAC motifs, and *PpTCP1*, *PpTCP13*, and *PpTCP15* can activate the expression of *PpTPS1*, as verified by Y1H and dual-luciferase assays (Figure 5). Promoter analysis revealed that all three genes contained ABRE *cis-*elements (Table 1). A qRT-PCR analysis revealed that they could all be activated by ABA, and analysis of their expression patterns in response to ABA with *PpTPS1* revealed that *PpTCP1* was significantly expressed in the early stages of ABA treatment, similar to the expression patterns of *PpTPS1* in response to ABA, drought, and salt stress. *PpTCP13* was expressed at the late stage of ABA treatment, while *PpTCP15* had two expression peaks, presumably regulating the *PpTPS1* and ABA signaling pathways in different ways.

## Conclusion

In summary, *PpTCP1*, *PpTCP13*, and *PpTCP15* were induced by ABA to activate *PpTPS1* expression, while *PpTPS1* was able to interact with *PpABI5* to participate in the ABA regulatory pathway, and it is speculated that *PpTPS1* could play a role in the regulation of salt acclimation in plants.

## Data Availability Statement

The original contributions presented in the study are included in the article/Supplementary Material, further inquiries can be directed to the corresponding authors.

## Author Contributions

LL, XC, and XM designed the study. XM, NW, BW, RZ, and HH performed the experiments and analyzed the data. XM wrote the manuscript. All authors contributed to the article and approved the submitted version.

## Conflict of Interest

The authors declare that the research was conducted in the absence of any commercial or financial relationships that could be construed as a potential conflict of interest.

## Publisher’s Note

All claims expressed in this article are solely those of the authors and do not necessarily represent those of their affiliated organizations, or those of the publisher, the editors and the reviewers. Any product that may be evaluated in this article, or claim that may be made by its manufacturer, is not guaranteed or endorsed by the publisher.
